# The economic burden of chronic non-communicable diseases in rural Malawi: an observational study

**DOI:** 10.1186/s12913-016-1716-8

**Published:** 2016-09-01

**Authors:** Qun Wang, Stephan Brenner, Olivier Kalmus, Hastings Thomas Banda, Manuela De Allegri

**Affiliations:** 1 Institute of Public Health, University of Heidelberg, INF 324, 69120 Heidelberg, Germany; 2 Faculty of Humanities and Social Sciences, Dalian University of Technology, Linggong Road No. 2, Ganjingzi District, Dalian, China; 3 Research for Equity and Community Health Trust (REACH Trust), P.O. Box 1597, Lilongwe, Malawi

**Keywords:** Chronic non-communicable diseases, Economic burden, Malawi, Catastrophic health expenditure

## Abstract

**Background:**

Evidence from population-based studies on the economic burden imposed by chronic non-communicable diseases (CNCDs) is still sparse in Sub-Saharan Africa. Our study aimed to fill this existing gap in knowledge by estimating both the household direct, indirect, and total costs incurred due to CNCDs and the economic burden households bear as a result of these costs in Malawi.

**Methods:**

The study used data from the first round of a longitudinal household health survey conducted in 2012 in three rural districts in Malawi. A cost-of-illness method was applied to estimate the economic burden of CNCDs. Indicators of catastrophic spending and impoverishment were used to estimate the economic burden imposed by CNCDs on households.

**Results:**

A total 475 out of 5643 interviewed individuals reported suffering from CNCDs. Mean total costs of all reported CNCDs were 1,040.82 MWK, of which 56.8 % was contributed by direct costs. Individuals affected by chronic cardiovascular conditions and chronic neuropsychiatric conditions bore the highest levels of direct, indirect, and total costs. Using a threshold of 10 % of household non-food expenditure, 21.3 % of all households with at least one household member reporting a CNCD and seeking care for such a condition incurred catastrophic spending due to CNCDs. The poorest households were more likely to incur catastrophic spending due to CNCDs. An additional 1.7 % of households reporting a CNCD fell under the international poverty line once considering direct costs due to CNCDs.

**Conclusion:**

Our study showed that the economic burden of CNCDs is high, causes catastrophic spending, and aggravates poverty in rural Malawi, a country where in principle basic care for CNCDs should be offered free of charge at point of use through the provision of an Essential Health Package (EHP). Our findings further indicated that particularly high direct, indirect, and total costs were linked to specific diagnoses, although costs were high even for conditions targeted by the EHP. Our findings point at clear gaps in coverage in the current Malawian health system and call for further investments to ensure adequate affordable care for people suffering from CNCDs.

**Electronic supplementary material:**

The online version of this article (doi:10.1186/s12913-016-1716-8) contains supplementary material, which is available to authorized users.

## Background

Chronic non-communicable diseases (CNCDs), defined as non-communicable conditions that affect people in the long term [1], account for the vast majority of deaths and are responsible for a notable economic burden in low- and middle-income countries (LMICs). It has been estimated that in 2010 alone, the economic loss due to CNCDs amounted to 500 billion US Dollar (USD) in LMICs, accounting for 4 % of these countries’ GDP [2].

The World Health Organization (WHO) predicts that by 2030 the largest proportional rise in CNCD deaths globally will occur in Sub-Saharan Africa (SSA), where the disease burden of CNCDs is already very large [3]. Considering the paucity of adequate social protection structures in most SSA countries [4], the increasing economic burden due to CNCDs is likely to become ‘catastrophic’ and aggravate poverty among local communities, who already struggle to meet basic daily needs [5].

Still, compared with communicable diseases, researchers have paid limited attention to the economic burden imposed by CNCDs in SSA [6]. The most relevant evidence on the economic burden related to CNCDs comes from high-income countries (HICs) [7]. In SSA, the few studies on costs associated with CNCDs used facility-based convenience samples from patients diagnosed with a specific CNCD [8–11] or relied on various secondary data sources [12] or on projections derived from assumptions of data and models first used in other settings [13]. Evidence from population-based studies specifically aimed at exploring the overall economic burden imposed by CNCDs, especially by a broad range of CNCDs, is still sparse in these settings. In addition, to our knowledge, no study has been conducted to assess the economic impact of CNCDs specifically on affected households in SSA. The few existing studies on catastrophic spending and impoverishment in SSA used CNCDs as one of explanatory variables influencing the odds of catastrophic spending and impoverishment [14, 15]. Evidence on the economic impact of CNCDs specifically on affected households exists from other settings in LMICs [16–18]. However, given remarkable disparities in overall social and political settings, the available evidence cannot provide direct policy guidance for SSA governments.

This population-based study was designed to fill this existing gap in knowledge by describing direct, indirect, and total costs for the most frequently reported CNCDs and the related economic impact of these conditions on affected households in rural Malawi.

## Methods

### CNCDs and health service provision in Malawi

In recent years, CNCDs have been causing an increasing number of deaths and disabilities in Malawi [19]. In 2008, CNCDs were estimated to account for 28 % of all deaths [20], ranking second to HIV/AIDS as the most prevalent cause of death in adults [21].

Health service provision in Malawi relies on a mixture of public and private providers. Government funded health provision consists of a combination of tax-based and donor financing to ensure that services included in the Essential Health Package (EHP) are provided free of charge at point of use, at either public or contracted private not-for-profit facilities. The EHP is meant as a measure to advance progress towards universal coverage and as such includes most priority health interventions targeting the major causes of disease burden in the country [22]. In 2010, the government expanded the EHP to also include health interventions targeting the most common CNCDs, such as screening, prevention, and treatment of cardiovascular diseases, diabetes, and certain cancers (breast cancer, cervical cancer) [21]. As shown in our previous work, however, health service utilization by people suffering from any of these CNCDs remains rather poor [23].

### Data and data collection

For this study, we used data from the first round of a panel household survey conducted from August to October 2012 in the districts of Thyolo, Chiradzulu, and Mulanje in Southern Malawi. These districts count a population of 1.4 million and include a total of 76 health facilities offering the EHP (60 public facilities and 16 private not-for-profit facilities). In addition, there are 6 private for-profit facilities. Details of the sampling procedures for the panel household survey have been described elsewhere [23]. In brief, given that the ultimate purpose of the panel was to evaluate the impact of a micro-health insurance scheme to be launched by the local Savings and Credit Cooperative (SACCO), the panel included both households where at least one person was a SACCO member (*n* = 691) and households where no person was a SACCO member (*n* = 525). The total sample included 5643 individuals distributed in 1199 households. The panel survey questionnaire collected information on both household socio-demographic and economic characteristics, on individual illness profiles, and on the specific health seeking behavior (including costs) of people suffering from acute and chronic conditions (the relevant sections of the household survey questionnaire are included in Additional file 1).

People suffering from at least one CNCD were identified in two steps. First, we asked respondents whether they suffered from any chronic disease, which we defined as any illness or complaint that persisted in an individual longer than three months, or any recurrent non-communicable illness episodes that had initially started in the past and continued to affect a respondent’s health by the time of interview [24]. In a second step, we collected information on diagnoses and/or symptoms and recorded both clinical terminology and lay language from all people reporting at least one chronic complaint in order to further classify the reported chronic complaints. Lists of diagnoses and symptoms were used to facilitate data collection. Respondents were given the option to record more than one chronic condition. Using a simplified algorithm, each reported diagnosis or symptom complex was assigned to one of ten WHO categories for non-communicable conditions [3].

Once identified as suffering from a chronic condition, individuals were asked a series of additional questions pertaining to their health seeking behavior and related direct and indirect costs. In consonance with other researches [25–29], in order to minimize recall bias, we used a four-week recall period to elicit responses on the reported CNCD health seeking behavior and costs.

### Variables and their measurements

We defined the primary outcome variables for this study as the direct and indirect costs as well as the sum of these two costs, i.e. total costs, incurred by an individual due to CNCDs. All cost values are expressed in the local currency, Malawian Kwacha (MWK) (1USD ~ 280MWK at the time of survey). In addition, based on the analysis of direct costs only, we looked at catastrophic spending and impoverishment due to CNCDs.

#### Direct costs

We defined direct costs as the out-of-pocket expenditure arising in the process of seeking care, including direct medical and direct non-medical costs [30]. In this study, direct medical costs included consultation fees, laboratory tests, drugs, medical aids, and disposables, while direct non-medical costs included only transport costs directly associated with all care-seeking events incurred over the one-month recall period.

#### Indirect costs and total cost of illness

In line with prior research [31] using the human capital approach (a most commonly used method of calculating indirect cost in cost-of-illness studies, which estimates the value of human life as the value of the output produced by the individual over his/her lifetime expressed as a function of the individual’s earnings [32]), we estimated indirect costs as the value of lost work time over the set recall period due to CNCDs among the economically active persons (10–64 years) reporting such a condition. Specifically, to estimate indirect costs and in line with prior research [33] conducted in rural SSA, we multiplied the country-specific minimum wage for 2012, 317 MWK per day [34], by the lost work time reported by respondents as a consequence of CNCDs. Our questionnaire did not include questions to allow us to estimate the opportunity costs incurred by caretakers of people reporting a CNCD. Therefore, we could not include such an estimate in our calculations. Individuals younger than 10 years or older than 64 years were deemed to be economically non-active. Consistent with a previous study conducted in Ethiopia [31], direct costs were calculated only for those individuals who had sought some form of care, while indirect costs were estimated for all individuals aged 10 to 64 years, independently of whether they had sought care or not.

The total cost of illness due to CNCDs was estimated by simply adding direct and indirect costs for all individuals who reported at least one CNCD. When calculating total costs, direct costs for those who did not seek any care and indirect costs for those who were younger than 10 years or older than 64 years were counted as zero [31].

#### Catastrophic spending

We defined catastrophic spending due to CNCDs as the share of household out-of-pocket health expenditure caused by CNCDs in relation to household non-subsistence expenditure (total household expenditure minus expenditure for basic subsistence needs) once exceeding a given threshold level [35]. In practice, in line with previous studies [14, 35–37], we used food expenditure as a proxy of a household’s basic subsistence needs. Also in line with prior research [36], we set the threshold levels in this study were set at 10 %, 25 %, and 40 %.

#### Impoverishment

We defined impoverishment due to CNCDs as the difference in poverty rate before paying for CNCD care (i.e. pre-payment poverty rate) and after making such payments (i.e. post-payment poverty rate), i.e. the percentage of non-poor households falling into poverty due to direct costs associated with CNCDs [38, 39]. Due to the fact that information on the national poverty line for 2012 was not available in Malawi, we used the international poverty line, $1.25 per person per day [40]. We converted the $1.25 per person per day into the local currency using the official 2012 purchasing power parity exchange rate of 88.17 [41]. This yielded a poverty line of 3306.4 MWK per person per month.

#### Analytical approach

Due to the uneven distribution of direct, indirect, and total costs, we used mean, median, and inter-quartile range to describe the central tendency of each of these cost estimates. The incidence of catastrophic spending due to CNCDs was computed as the proportion of households incurring catastrophic spending in relation to the total number of households reporting CNCDs [38, 42]. We also analyzed the distribution of the incidence of catastrophic spending across social-economic quartiles. The incidence of impoverishment due to CNCDs was calculated in a similar way, i.e. the proportion of households being impoverished in relation to the total number of households reporting CNCDs [38, 42]. The four-week per capita household expenditure was used as a proxy of social-economic status (SES) [24, 28, 43–45]. Per capita household expenditure was computed by dividing total household expenditure by the number of people living in a household. In this study, total household expenditure included items on food, alcohol, clothing, housing, transportation, communication, entertainment, education, personal care, insurance, transfers remittances, and health care (acute and chronic conditions) spending.

## Results

Out of 5643 individuals included in the survey, 475 (8.4 %) reported a total of 515 chronic non-communicable conditions (i.e. among these 475 individuals, on average, each respondent reported 1.08 CNCDs). Out of the ten disease categories used in our study to group chronic complaints, the six most frequently reported were: chronic pains (*n* = 126, accounting for 24.5 % of all reported conditions), chronic respiratory conditions (*n* = 98, 19.0 %), chronic cardiovascular conditions (*n* = 95, 18.4 %), chronic sense organ conditions (*n* = 65, 12.6 %), chronic neuropsychiatric conditions (*n* = 47, 9.1 %), and chronic digestive conditions (*n* = 42, 8.2 %). Chronic skin or oral conditions, malignant neoplastic conditions, chronic genitourinary conditions, and chronic endocrine conditions were excluded from further analysis due to the small number of respondents in these categories. Among the six most frequently reported conditions, only chronic cardiovascular conditions were covered by the EHP at the time of the study. Out of these 475 respondents reporting at least one CNCD, 298 (62.7 %), distributed in 244 households, sought some form of either formal (services provided by public facilities, private not-for-profit facilities, and private for-profit facilities) or informal care (traditional care and self-treatment) during the four weeks preceding the interview, while 320 (67.4 %) were of 10 to 64 years of age.

### Cost of illness

Among the 298 individuals who sought care for CNCDs, mean monthly direct costs were 854.82MWK, with direct medical costs accounting for 75 % of direct costs. Mean monthly direct costs were highest for individuals seeking care for chronic neuropsychiatric and chronic cardiovascular conditions, at 1,677.60 MWK (73.8 % medical costs) and 1,617.83 MWK (60.6 % medical costs) respectively. Mean monthly direct costs were lowest for individuals with chronic sense organ conditions and chronic digestive conditions, at 410.31 MWK (82.5 % medical costs) and 416.38 MWK (80.5 % medical costs), respectively (Table 1).

**Table 1:** Direct cost of CNCDs of individuals who sought care in MWK

	Mean	Median	Inter-quartile range	Direct medical cost as % of direct cost of illness
Total CNCD sample (*n* = 298)	854.82	150	(500, 0)	75.0 %
Chronic pain conditions (*n* = 89)	464.83	200	(400, 0)	79.0 %
Chronic respiratory conditions (*n* = 56)	530.89	50	(600, 0)	72.5 %
Chronic cardiovascular conditions (*n* = 60)	1617.83	150	(810, 0)	60.6 %
Chronic sense organ conditions (*n* = 32)	410.31	150	(500, 15)	82.5 %
Chronic neuropsychiatric conditions (*n* = 25)	1677.60	450	(1000, 100)	73.8 %
Chronic digestive conditions (*n* = 29)	416.38	150	(300, 0)	80.5 %

Among the 320 individuals aged 10 to 64 years reporting CNCDs, mean monthly indirect costs were 748.91 MWK with an average of 2.36 lost days per month due to CNCDs. Among the six most frequently reported CNCDs, those who reported chronic digestive conditions, chronic neuropsychiatric conditions, and chronic cardiovascular conditions bore the highest level of mean monthly indirect costs (929.14 MWK, 898.17 MWK, and 833.14 MWK respectively), while those who reported chronic sense organ conditions and chronic respiratory conditions bore the lowest mean monthly indirect costs (503.47 MWK and 627.66 MWK, respectively) (Table 2).

**Table 2:** Indirect cost of individuals aged 10 to 64 years reporting at least one CNCD

	Indirect cost (MWK)			Lost days due to CNCD (days)		
	Mean	Median	Inter-quartile range	Mean	Median	Inter-quartile range
Total CNCD sample (*n* = 320)	748.91	0	(951,0)	2.36	0	(3,0)
Chronic pain conditions (*n* = 95)	784.16	0	(951,0)	2.47	0	(3,0)
Chronic respiratory conditions (*n* = 50)	627.66	0	(951,0)	1.98	0	(3,0)
Chronic cardiovascular conditions (*n* = 78)	833.14	0	(1268,0)	2.63	0	(4,0)
Chronic sense organ conditions (*n* = 34)	503.47	0	(951,0)	1.59	0	(3,0)
Chronic neuropsychiatric conditions (*n* = 30)	898.17	0	(951,0)	2.83	0	(3,0)
Chronic digestive conditions (*n* = 29)	929.14	0	(1268,0)	2.93	0	(4,0)

Mean monthly total costs, including both direct and indirect costs, were 1,040.82 MWK, of which 56.8 % was contributed by direct costs. Mean monthly total costs were highest for individuals reporting chronic cardiovascular conditions and chronic neuropsychiatric conditions (1,723.99 MWK and 1,465.64 MWK respectively) and lowest for individuals reporting chronic sense organ conditions and chronic respiratory conditions (465.35 MWK and 623.60 MWK respectively) (Table 3).

**Table 3:** Total cost of CNCDs of all individuals reporting at least one CNCD in MWK

	Mean	Median	Inter-quartile range	Direct cost as % of total cost of illness
Total CNCD sample (*n* = 475)	1040.82	40	(1000, 0)	56.8 %
Chronic pain conditions (*n* = 126)	926.92	200	(1268, 0)	51.6 %
Chronic respiratory conditions (*n* = 98)	623.60	0	(900, 0)	60.8 %
Chronic cardiovascular conditions (*n* = 95)	1723.99	200	(1600, 0)	54.0 %
Chronic sense organ conditions (*n* = 65)	465.35	0	(357, 0)	66.4 %
Chronic neuropsychiatric conditions (*n* = 47)	1465.64	50	(951, 0)	65.2 %
Chronic digestive conditions (*n* = 42)	929.05	70	(1268, 0)	53.3 %

### Economic impact of CNCDs

Table 4 presents the incidence of catastrophic spending due to CNCDs at the household level. Out of 244 households with at least one household member reporting a chronic non-communicable illness and seeking care for such a condition, 21.3 % reported that direct costs due to their chronic illness exceeded 10 % of household non-food expenditure. Increasing the threshold to 25 % and 40 % reduced the incidence of catastrophic spending to 10.7 % and 4.5 %, respectively. The incidence of catastrophic spending was different across SES quartiles with the poorest households being more likely to incur catastrophic spending due to CNCDs.

**Table 4:** Incidence of household catastrophic spending due to CNCDs

	10 %	25 %	40 %
1st Quartile (lowest SES) (*n* = 51)	35.3 %	23.5 %	9.8 %
2nd Quartile (*n* = 44)	20.5 %	11.4 %	4.5 %
3rd Quartile (*n* = 65)	23.1 %	7.7 %	3.1 %
4th Quartile (highest SES) (*n* = 84)	11.9 %	4.8 %	2.4 %
Total (*n* = 244)	21.3 %	10.7 %	4.5 %

Table 5 presents household impoverishment due to CNCDs. Out of 244 households with at least one household member reporting a chronic non-communicable condition and seeking care for such a condition, 43.0 % already lived under the international poverty line of $1.25 per person per day before expenses in form of any direct out-of-pocket payment due to CNCDs occurred. Once adjusted for spending on care for CNCDs, impoverishment increased to 44.7 % in respect to the international poverty line.

**Table 5:** Household impoverishment due to CNCDs (*n* = 244)

Pre-payment poverty rate	Post-payment poverty rate
43.0 %	44.7 %

## Discussion

To our knowledge, this is one of the very first studies exploring the economic burden imposed on SSA households by a broad range of CNCDs, with information derived from population-based rather than a facility-based sample. Our findings indicate that CNCDs carry a relatively high economic burden for the local population, which causes financial stress and aggregates poverty in rural Malawi. In addition, our study identified remarkable differences in economic burden depending on the specific condition affecting an individual. Considering the scarcity of cost-of-illness studies on CNCDs in SSA, we discuss our findings in relation to literature emerging from other LMICs as well.

Our study found that direct costs accounted for the largest portion of the total cost of illness across a broad range of CNCDs, whereas direct medical costs comprised the largest portion of direct costs to the individual as a result of these conditions. These findings are consistent with cost-of-illness studies on cervical cancer [8] and on heart failure [10] using facility-based data in SSA and studies on hypertension [16] and on diabetes [46] relying on population-based surveys from other LMICs. These findings suggest that in Malawi, where care is in principle free of charge at point of use, people still bear an important burden due to direct costs caused by CNCDs. In line with a prior qualitative study looking at people’s perceptions of gaps in coverage [47], our findings confirm that the officially free health system fails to offer adequate financial protection against direct costs due to CNCDs.

Also in line with prior evidence [8, 10, 16, 46], when computing total cost of illness, indirect costs were found to be of comparably high magnitude as direct costs. To appreciate the magnitude of the indirect costs, one needs to assess them against the value of the existing poverty line. Moreover, one needs to consider that, given our inability to value productivity losses by caregivers, we might have underestimated the actual magnitude of the productivity loss associated with CNCDs. In addition, one needs to consider that productivity losses in the SSA bear important consequences for households’ economic well-being, since social health protection mechanisms are not sufficiently developed to provide proper compensation for productivity losses due to illness. Thus, productivity losses are born by ill people themselves [48], since inability to work translates into actual wealth losses. This represents an important gap in social health protection, which adds to the burden imposed by direct out-of-pocket spending and deserves to be addressed equally urgently by local policy makers.

Among the most frequently reported CNCDs, we showed that individuals affected by chronic cardiovascular conditions and chronic neuropsychiatric conditions bore the highest levels of direct, indirect, and total costs. This finding is in line with the report on the global economic burden of non-communicable diseases recently released by Harvard University [49]. Also, chronic neuropsychiatric conditions, such as depression, anxiety, or mental disorders, are currently not yet addressed by the EHP in Malawi. In the light of our findings reporting the importance of the economic burden associated with these conditions, policy makers should consider expanding coverage through the EHP since an expansion in service coverage could translate into increased financial protection for the local population.

The fact that chronic cardiovascular conditions resulted in the highest level of direct costs appears even more striking, given that these conditions are covered by the EHP and related services should be provided free of charge at point of use. Again in line with prior qualitative evidence [47], our findings suggest that policy does not translate into effective coverage so that gaps in service coverage and financial protection remain very high, even for services explicitly targeted by the EHP.

Our findings also revealed that a considerable proportion of households incurred catastrophic spending due to CNCDs. Poorest households faced the highest risk of catastrophic spending due to CNCDs. These findings, which rely on direct costs for CNCDs aggregated at the household level, support findings from our previous work, which pointed at the regressivity of individual out-of-pocket expenditure on CNCDs [45]. These findings are also consistent with prior analyses of catastrophic spending, which included chronic conditions as an explanatory variable in models targeting the overall economic burden due to ill health in SSA [14, 15] and in LMICs more in general [28, 39, 42, 50, 51]. These studies had already indicated how suffering from a chronic condition represented an important determinant of catastrophic spending.

In addition, our study found that an additional 1.7 % of households with at least one household member reporting a CNCD fell under the international poverty line once expenditures due to CNCDs were considered. The fact that the economic burden of CNCDs aggravates poverty is in line with previous studies focusing specifically on the economic impact of CNCDs on affected households [16, 18] and using CNCDs as explanatory variable influencing the incidence of impoverishment [15, 39, 42, 51] in LMICs. In the context of Malawi, this finding appears worrisome in light of the rapid growth of CNCDs and the fact that above 40 % of households already live below the international poverty line.

### Methodological considerations

At last, we need to consider a few limitations of our study. First, as mentioned in the methods section, we could not estimate caretakers’ productivity losses, due to absence of relevant information from the survey. In addition, our estimation of direct costs is likely to be lower than their true value, since we did not have data on other direct non-medical costs, such as food and accommodation expenses incurred by the sick individuals and their caretakers. Thus, both direct and indirect costs resulting in the overall economic burden are likely to be higher than what estimated in our study. Second, the individual’s earnings are not easily traceable in subsistence societies where people produce most of what they will consume [52]. This represents the main challenge in applying the human capital approach to estimate indirect costs in these settings. In line with previous research [33], we used minimum wage as a proxy of the individual’s earnings in the informal sector. We need to be aware that our study could have led to somewhat different estimates of indirect cost if we had used different proxies of one’s earnings (still based on the human capital approach) to estimate productivity losses, such as the per capita Gross Domestic Product [53], per capita expenditure [54], and the marginal product of labor [55]. Furthermore, we need to consider that alternative methods of assessing productivity losses, not based in the human capital approach, such as willingness-to-pay could have potentially generated higher estimates than ours [52, 56]. This can be explained in relation to the fact that willingness-to-pay generates etimates based on a series of hypothetical questions. As such, the method may induce respondents to provider higher estimates than what they would actually be willing to pay in reality [52]. Third, both direct costs and productivity losses were estimated based on information reported directly by the individuals and may therefore be subject to recall bias. Like in many other rural settings in SSA, it was not possible for us to validate the veridicity of the information provided by the respondents, given the absence of formal medical booklets documenting illness and healthcare seeking. The use of a four-week recall period, rather than a longer recall period as applied in other CNCD studies [24, 46, 57–59], was designed to minimize recall bias [58, 60]. Still, in line with prior research [61, 62], we cannot exclude that in spite of our efforts to minimize recall bias, we did not to some extent underestimate or overestimate direct and indirect costs. Underestimation of direct costs might have occurred as the result of the fact that individuals may incur direct expenditure related to CNCD on larger time intervals and may have therefore not spent anything in the prior four weeks, because still in possession of enough medications to control their condition from a prior prescription. On the contrary, overestimation of indirect costs might have occurred as a result of potential over-reporting time loss due to illness. Forth, since our study represents an initial exploratory effort relying on population-based data to estimate the economic burden imposed by CNCDs in SSA, we did not have capacity to integrate measures of intangible costs (e.g. the level of pain or suffering due to specific health condition). Further studies are needed to explore the intangible costs associated with suffering from a CNCD in a SSA setting. Fifth, the methodology currently available to estimate catastrophic spending and impoverishment does not account for people who forego care, since these individuals do not report any out-of-pocket expenditure due to illness [38]. Households forgoing treatment are usually the poorest in a community. Forgoing treatment causes further deterioration of one’s health, often leading to further economic losses and deeper poverty. Thus, the real economic impact of CNCDs is likely to be much higher than what can be estimated using available methodology and what we were able to show in our study, using catastrophic spending and impoverishment as relevant indicators [36, 38].

## Conclusion

Our study showed that in rural Malawi, the economic burden of CNCDs remains high, leading households to face catastrophic spending and pushing households further into poverty. This finding is worrisome given that in Malawi, health care services are set to be provided free of charge at point of use for a broad range of conditions, including several chronic non-communicable ones. Our findings further indicated that particularly high direct, indirect, and total costs were linked to specific diagnoses, but that in general costs were high even for conditions in principle covered by the EHP. These findings point at clear gaps in financial protection and call for further investments, not only increasing effective coverage of chronic conditions via the EHP, but also expanding formal social health protection mechanisms, such as offering monetary compensation for work lost in case of illness. Further studies are needed to replicate and thus validate our research findings in other SSA settings.

## Additional file

Additional file 1: The questionnaire for CNCDs. Data description: the section for CNCDs in household survey questionnaire in our study is presented in the file. (DOCX 31 kb)

## Abbreviations

CNCDs: Chronic non-communicable diseases
EHP: Essential health package
HICs: High-income countries
LMICs: Low- and middle-income countries
MWK: Malawian kwacha
SACCO: Savings and credit cooperative
SES: Social-economic status
SSA: Sub-Saharan Africa
USD: US dollar
WHO: World Health Organization
