# Supplementary material for: The economic burden of chronic non-communicable diseases in rural Malawi: an observational study
Source: BMC Health Serv Res. 2016 Sep 1;16(1):457. doi: 10.1186/s12913-016-1716-8 (PMC5007731; doi:10.1186/s12913-016-1716-8)
Supplement: Additional file 1: — The questionnaire for CNCDs. Data description: the section for CNCDs in household survey questionnaire in our study is presented in the file. (DOCX 31 kb) [file 12913_2016_1716_MOESM1_ESM.docx]

## Additional file 1: The questionnaire for CNCDs

In this section we would like to ask you about chronic illnesses. By chronic we mean any illness which you have that has lasted longer than three months or any illness that came up earlier in your life and has not disappeared until today.

| 1 | Do you suffer from any of these chronic conditions (Please make sure to enter only chronic conditions):  (If the respondent answer ‘No’ to any of these chronic conditions, please go to acute section) | Yes or No | If yes, for how many years have you had these chronic symptoms? |
| --- | --- | --- | --- |
|  | Longstanding problems with lungs or breathing / Chronic respiratory infections or lung diseases (e.g. a. Asthma (wheezing attacks), b. Chronic bronchitis/emphysema/COPD (chronic productive cough and wheezing), c. Pulmonary fibrosis/sarcoidosis (dry cough and shortness of breath)) |  |  |
|  | Chronic high blood pressure / hypertension |  |  |
|  | Longstanding problem with the heart or blood circulation / cardiovascular diseases (e.g. a. Coronary artery disease (intermittent right-sided radiating chest pain), b. Dysrhythmias (irregular heart beats), c. Heart failure (oedema, shortness of breath), d. Inflammatory heart disease/rheumatic fever, e. Sequelae of brain stroke (focal weakness, abnormal speech)) |  |  |
|  | Longstanding problems with the mood, chronic mental or psychiatric conditions (e.g. a. Depression, b. Longstanding anxiety/paranoia/PTSD, c. Dementia, Alzheimer d. Schizophrenia / psychotic disorder (often referred to as "being crazy"), e. Epilepsy / seizure disorder, f. Mental retardation / brain damage, cerebral palsy (meaning mentally handicapped since birth/childhood) g. Speech impediment) |  |  |
|  | Cancer, malignant tumor or neoplasms (e.g. Lung cancer, Stomach/oesophagous cancer, Liver cancer, Colorectal cancer, Breast cancer, Cervical/uterine cancer, Leukemia, Brain tumor, Lymphoma) |  |  |
|  | Raised blood sugar or Diabetes mellitus |  |  |
|  | Longstanding diseases of the stomach, bowel or the liver / chronic digestive diseases (e.g. a. Chronic diarrhoea/ malabsorption/ tropical sprue/ colitis/ Morbus Crohn, b. Heartburn / reflux disease, upset stomach/ gastritis / PUD, c. rectal prolapse / hemorrhoids(piles), d. Hepatitis / cirrhosis of liver / jaundice, e. gall stones / cholecystitis, f. Hernia) |  |  |
|  | Longstanding/chronic pains / aches (e.g. a. Recurrent headaches / migraine, b. Chronic back pain, c. joint pain / arthrosis, d. Arthritis / rheumatism, e. sickle cell) |  |  |
|  | Physical handicap, deformities or disabilities (longstanding problems with mobility, limb function (bones, legs, arms) (e.g. a. Difficulty walking / unable to walk, b. Missing / non-functional / stiff limbs) |  |  |
|  | Longstanding problems with the eyes, ears or nose: e.g. a. poor eyesight / blindness, b. Chronic conjunctivitis, c. Trachoma, e. Cataract, f. Poor hearing / deafness g. Chronic sinusitis |  |  |
|  | Longstanding problems with the skin / Dermatologic Diseases: (e.g. Ectopic Dermatitis, Gangrene, Guinea worm, Ringworm, Scabies) |  |  |
|  | Any other chronic diseases or other permanent diseases (lasting longer than the last three months)  (If Yes, please go to question 2. If No, please go to question 3) |  |  |

| 2 | Could you please describe the health problem(s) you had. (symptoms, syndromes).  (please see symptom code and write down the code of symptom/symptoms answered by the respondent) |  |
| --- | --- | --- |
|  | symptom or syndrome 1 |  |
|  | symptom or syndrome 2 |  |
|  | symptom or syndrome 3 |  |
|  | symptom or syndrome 4 |  |

| Symptom code | |
| --- | --- |
| General Symptoms  1. Dehydration / Fluid loss  2. Fainting / Passing out  3. Fatigue / Lethargy  4. Fever / Chills  5. Loss of appetite / Unable to breast feed  6. Night sweats  7. Swelling feet / legs / body  8. Weight loss  Skin:  11. Boils / Abscess on skin  12. Itching  13. Jaundice / Yellow eyes  14. Pallor  15. Skin rash / blisters / localized swelling  16. Skin sore / Ulcer / Gangrene  Head & Face:  21. Congested / runny nose  22. Ear pain  23. Eye pain / tearing / redness  24. Facial rash / Swelling of face  25. Headache  26. Hearing loss  27. Jaw-lock  28. Nose bleeding  29. Sore Throat / Difficulties swallowing  30. Toothache / Sore mouth  31. Vision loss  Mental Status:  41. Abnormal behaviour / speech / cognition  42. Anxiety  43. Confusion  44. Convulsions  45. Dizziness / Spinning sensation  46. Insomnia / Sleeplessness  47. Loss of consciousness  48. Sadness  49. Suicidal ideation  50. Addiction (alcohol, drugs, tobacco) | Neck & Back:  51. Back / neck pain  52. Neck swelling  53. Stiff Neck  Chest & Respiration:  61. Cough (dry / with sputum / with blood)  62. Chest pain / tightness / pressure  63. Difficulty breathing / Shortness of breath / Wheezing  64. Rapid Heartbeat / palpitations  Abdomen & Digestion:  71. Abdominal / Pelvic / Flank pain or cramping  72. Anal / rectal pain  73. Blood in vomit / stool / urine  74. Constipation  75. Diarrhoea  76. Nausea / vomiting  77. Stomach pain / Heartburn  Urinary Tract & Genitals:  81. Abnormal vaginal bleeding  82. Frequent urination  83. Menstrual cramps / pain  84. Painful / burning urination / Dark urine  85. Urine / Stool incontinence  86. Vaginal / Penile discharge / sores / ulcers  87. Vaginal bleeding / Painful menstruation  Muscles, Bones & Joints:  91. Joint pain / Bone pain  92. Joint swelling  93. Muscle soreness / pain (myalgia)  94. Paralysis / Palsy  Pregnancy / Delivery / Newborn:  96. Prolonged labour  97. Prolonged bleeding after delivery / postnatal hemorrhage  98. Pain / Vaginal bleeding during pregnancy |

| 3 | Who did you consult for treatment for this/these chronic condition(s) during the last four weeks?  *(1) Health care provider (western medicine); (2) Community health worker or community nurse; (3) Traditional healer or herbalist; (4) Myself/family members (self-treatment); (5) Did not seek treatment for this condition in the last four weeks*  (If the answer is (1), (2), and (3), please go to questions 4, 5, 7;  If the answer is (1) and (2), please go to questions 8, 9, 11, 17, 18;  If the answer is (1), please go to questions 12, 13, 14, 15, 16;  If the answer is (3), (4), and (5), please go directly to question 20) |  |
| --- | --- | --- |
| 4 | The treatment(s) for this/these chronic condition(s) in the last four weeks, was it a follow-up visit (e.g. for medication refill) or due to acute symptoms of the chronic condition or both?  *(1) Follow-up visit/check-up; (2) Acute symptoms; (3) Both* |  |
| 5 | During the last four weeks, were you supposed to take any drugs for this chronic condition?  *(1) Yes; (2) No*  (If Yes, please go to question 6; if No, please go to question 7) |  |
| 6 | Did you run out of the required drugs during the last four weeks?  *(1) Yes; (2) No* |  |
| 7 | At what point since these chronic symptoms occurred did you decide to seek treatment from a health care provider during the last four weeks?  *(1) Immediately; (2) Waited to see the severity of illness; (3) When it started affecting my day-to-day work; (4) When it started incapacitating me* |  |
| 8 | Where specifically did you get your treatment during the last four weeks?  *(1) General practitioner or clinic; (2) District hospital or other regional hospital; (3) Central hospital; (4) Mobile clinic; (5) Community health worker / village clinic; (6) Registered pharmacies for consultation and drugs; (8) Health centre; (99) Other* |  |
| 9 | What is the name of the facility?  (please see facility code and write down the code of facility answered by the respondent )  (If Other, please go to question 10) |  |

| Facility code | |
| --- | --- |
| 1. Bilal Dispensary (Chiradzulu)  2. Chipho Health Centre (Chiradzulu), CHAM  3. Chiradzulu District Hospital (Chiradzulu)  4. Chitera Health Centre (Chiradzulu)  5. Magomero Health Centre (Chiradzulu), CHAM  6. Mauwa Health Centre (Chiradzulu)  7. Mbulumbuzi Health Centre (Chiradzulu)  8. Milepa Health Centre (Chiradzulu)  9. Namadzi Health Centre (Chiradzulu)  10. Namitambo Health Centre (Chiradzulu)  11. Ndunde Health Centre (Chiradzulu)  12. Nkalo Health Centre (Chiradzulu)  13. PIM Health Centre (Chiradzulu), CHAM  14. St Joseph Hospital (Chiradzulu), CHAM  15. Bvumbwe BLM Clinic (Thyolo)  16. Bvumbwe Makungwa Maternity (Thyolo)  17. Bvumbwe Research Health Centre (Thyolo)  18. Changata Health Centre (Thyolo)  19. Chimaliro Health Centre (Thyolo)  20. Chimvu Maternity (Thyolo)  21. Chingadzi Community Hospital (Thyolo), CHAM  22. Gombe Maternity (Thyolo)  23. Hellena Oakley Health Centre (Mtambanyama) (Thyolo), CHAM  24. Khonjeni Health Centre (Thyolo)  25. Makapwa Health Centre (Thyolo), CHAM  26. Malamulo Hospital (Thyolo), CHAM  27. Mangunda Clinic (Maternity) (Thyolo)  28. Mapanga Clinic (Thyolo)  29. Mayaka Health Centre (Thyolo), CHAM  30. Mbalanguzi Dispensary (Thyolo), CHAM  31. Mikolongwe Health Centre (Thyolo)  32. Mitengo Health Centre (Thyolo)  33. Molere Health Centre (Thyolo)  34. Nkhataombere Maternity (Thyolo)  35. Nsabwe Dispensary (Thyolo) | 36. St Joseph Health Centre (Thyolo), CHAM  37. Thekerani Health Centre (Thyolo)  38. Thomas Health Centre (Thyolo), CHAM  39. Thyolo District Hospital (Thyolo)  40. Zoa Health Centre (Thyolo)  41. Mlambe Hospital (Blantyre), CHAM  42. Queen Elizabeth Central Hospital (Blantyre)  43. Mulanje Mission Hospital (Mulanje), CHAM  44. Pirimiti Community Hospital (Zomba), CHAM  45. St Lukes Hospital (Zomba), CHAM  46. Zomba Central Hospital (Zomba)  47. Bondo Health Centre (Mulanje)  48. Chambe Health Centre (Mulanje)  49. Chinyama Health Centre (Mulanje)  50. Chisitu Health Centre (Mulanje)  51. Chonde Health Centre (Mulanje)  52. Dzenje (Maternity) (Mulanje)  53. Kambenje Health Centre (Mulanje)  54. Mbiza Health Centre (Mulanje)  55. Milonde Health Centre (Mulanje)  56. Mimosa Dispensary (Mulanje)  57. Mpala Health Centre (Mulanje)  58. Mulanje BLM Clinic (Mulanje)  59. Mulanje District Hospital (Mulanje)  60. Mulanje Mission Hospital (Mulanje)  61. Mulomba Health Centre (Mulanje)  62. Muloza Health Centre (Mulanje)  63. Namasalima Health Centre (Mulanje)  64. Namphungo Health Centre (Mulanje)  65. Namulenga Health Centre (Mulanje)  66. Naphimba Health Centre (Mulanje)  67. Thembe Health Centre (Mulanje)  68. Thuchila Health Centre (Mulanje)  99. Other facility (please specify) |

| 10 | If other facility, please write the name of the facility (including the village/city) |  |
| --- | --- | --- |
| 11 | What was the most important reason for choice of this provider?  *(1) Referral; (2) Close proximity or easy to reach; (3) Better services; (4) Lower cost of service; (5) Shorter waiting time; (6) I have personal relations; (7) I received financial or in-kind incentives to go there; (8) Provider is contracted by medical insurance scheme; (9) I had no other option* |  |
| 12 | If you went to a health facility, was this health facility private, CHAM or Public?  *(1) Private; (2) CHAM; (3) Public; (98) Do not know* |  |
| 13 | If you went to a health facility, what was your main means of transport during the last four weeks?  *(1) Walking; (2) Public transport; (3) Hire taxi; (4) Cart; (5) Bicycle; (6) Motorbike; (7) Car; (8) By ambulance; (99) Other* |  |
| 14 | How many hours did it take you to get to the facility? |  |
| 15 | How many hours did you have to wait to be seen by the health personnel during the last four weeks? (beginning from the time you entered the facility) |  |
| 16 | During the last four weeks, have you had to stay in hospital for this/these chronic condition(s)?  *(1) Yes; (2) No* |  |
| 17 | If yes, how many nights did you sleep in the facility in total? |  |
| 18 | Overall, how satisfied were you with the services you received in the facility used?  *(1) very satisfied; (2) somewhat satisfied; (3) neither satisfied nor unsatisfied; (4) somewhat unsatisfied; (5) very unsatisfied* |  |
| 19 | Would you recommend the facility to family or friends?  (*1) Yes; (2) No* |  |
| 20 | Which reason best explains why you did not seek treatment at a health care provider (western medicine)?  (*1) Expensive medical treatment; (2) Minor complaints that don’t call for professional assistance; (3) Poor public health services; (4) Long wait to meet doctor; (5) No transport available; (6) Could not get time because of work or had other commitments; (7) Went to the facility but could not get public health care; (8) Family (spouse, in-laws) decided otherwise;*  *(99) Other* |  |
| 21 | Were any drugs given directly to you for free for this/these chronic condition(s) by the health staff treating you during the last four weeks?  *(1) Yes; (2) No* |  |
| 22 | Were any drugs prescribed for this/these chronic condition(s) by health staff to be obtained during the last four weeks? (inside or outside the facility)  *(1) Yes; (2) No*  (If Yes, please go to question 23; if Yes, please go to question 24) |  |
| 23 | Did you obtain all prescribed medicines?  (*1) Yes; (2) No*  (If Yes, please go to question 26; if No, please go to question 24 and 25) |  |
| 24 | How often did you not obtain the prescribed medicine during the last four weeks?  *(1) Never obtained the prescribed medicine; (2) Rarely obtained the prescribed medicine;*  *(3) Sometimes obtained the prescribed medicine; (4) Often obtained the prescribed medicine* |  |
| 25 | If prescribed drugs were not obtained, which reasons best explain why you did not obtain them?  *(1) Forgot to buy; (2) Drugs not available; (3) Used home made medicine instead; (4) Too expensive; (5) Did not have money; (6) Do not trust these drugs; (7) Felt no need to do so; (8) Negative side-effects of drugs; (99) Other* |  |
| 26 | How much did you have to pay for the following treatment items in relation to this/these chronic condition in the last four weeks? |  |
|  | All consultation and treatment costs for this/these condition(s), including: laboratory tests (X-rays etc.), drugs (tablets, injections, infusions, topical preparations etc.), medical devices (crutches, glasses, etc.), as well as informal fees. |  |
|  | Costs for traditional healers, herbalists, faith healers |  |
|  | Transportation (incl. Fuel) |  |
| 27 | How did you pay for all the health care expenditure that you incurred for this/these chronic condition(s) in the last four weeks?  *(1) I did not have to pay (services were for free); (2) Another institution paid all for me (e.g. insurer, NGO, employer); (3) Another institution paid something for me (e.g. insurer, NGO, employer) and I paid the rest out-of-pocket; (4) I paid everything out-of-pocket; (5) I was supposed to pay out of pocket but did not have the money at that moment and I am still expected to pay*  (If the answer is (2) or (3), please go to question;  If the answer is (3), (4), or (5), please go to question 29; |  |
| 28 | If another institution paid something/all for your treatment, what institution was it?  *(1) Employer; (2) Insurer; (3) NGO (incl. pre-paid voucher or cash incentives); (4) Government programme (incl. pre-paid voucher or cash incentives); (5) Churches/mosques etc.; (99) Other* |  |
| 29 | From which source (s) did you raise money to pay for these out-of-pocket payments? |  |
|  | Cash at home  *(1) Yes; (2) No* |  |
|  | Used SACCO savings  *(1) Yes; (2) No* |  |
|  | Used other bank savings  *(1) Yes; (2) No* |  |
|  | Used SACCO loans  (*1) Yes; (2) No* |  |
|  | Used other bank loans  *(1) Yes; (2) No* |  |
|  | Rotating savings and credit club (ROSCA)  *(1) Yes; (2) No* |  |
|  | Borrowed money from money lender  *(1) Yes; (2) No* |  |
|  | Borrowed money from friends/relatives  *(1) Yes; (2) No* |  |
|  | Assistance from friends and relatives (I don't have to pay it back)  *(1) Yes; (2) No* |  |
|  | Sold an asset  *(1) Yes; (2) No* |  |
|  | National remittances (member of family who is not living in household for more than three months)  *(1) Yes; (2) No* |  |
|  | International remittances  *(1) Yes; (2) No* |  |
|  | Other  *(1) Yes; (2) No* |  |
| 30 | Did your chronic conditions hinder you from carrying out routine activities?  *(1) Yes; (2) No*  (If Yes, please go to question 31 and 32; if No, please go to question 33) |  |
| 31 | How many days could you not carry out routine activities during the last four weeks due to these chronic conditions? |  |
| 32 | How many people (adults) took care of you throughout the course of treatment for these chronic conditions, i.e., from when you were unable to carry out routine activities until you got better? |  |
| 33 | Did you have to pay the people who took care of you throughout the course of treatment for these chronic conditions?  *(1) Yes; (2) No* |  |
| 34 | How much did you pay them to take care of you? |  |
| 35 | How many of these people who took care of you are female?  (If no less than 1 person took care of you, please go to question 36) |  |
| 36 | Did your chronic conditions prevent people that took care of you from working?  *(1) Yes; (2) No* |  |
